# Global conserved RBD fraction of SARS-CoV-2 S-protein with T500S mutation in silico significantly blocks ACE2 and rejects viral spike

**DOI:** 10.1186/s41231-022-00109-5

**Published:** 2022-02-04

**Authors:** Amrita Banerjee, Mehak Kanwar, Dipannita Santra, Smarajit Maiti

**Affiliations:** 1 Department of Biochemistry and Biotechnology, Cell and Molecular Therapeutics Laboratory, Oriental Institute of Science and Technology, Midnapore, India; 2 Department of Physiology, Raja Bazar Scienec College, Calcutta University, Calcutta, West Bengal India; 3 Agricure Biotech Research Society, Epidemiology and Human Health Division, 721101 Midnapore, India

**Keywords:** SARS CoV-2 pandemic, ACE2 blocking by RBD fragments, Mutation in RBD, T500S, Haddock and Hawkdock

## Abstract

**Background:**

SARS-CoV-2 developed global-pandemic with millions of infections/deaths. As it is urgently necessary it is assumed that some blockers/inhibitors of ACE2 could be helpful to resist the binding of viral-spike Receptor-Binding-Domain (RBD).

**Methods:**

Here, conserved RBD from 186-countries were compared with WUHAN-Hu-1 wild-type (CLUSTAL-X2/Pymol). The RBD of ACE2-bound nCOV2 crystal-structure 6VW1 was analyzed by Haddock-PatchDock. Extensive structural study/trial to introduce point/double/triple mutations in the different locations of CUT4 (most-effective from total 4 proposed fragments; CUTs) were tested with Swiss-Model-Expacy.

**Results:**

Blind-docking of mutated-CUTs in ACE2 completely rejected the nCOV2 binding to ACE2. Further, competitive-docking/binding-analyses (by PRODIGY) demonstrated few more bonding (LYS31-PHE490 and GLN42-GLN498) of CUT4 (than wild) and hindered TYR41-THR500 interaction with ACE2. Moreover, mutated-CUT4 even showed higher blocking effect against spike-ACE2 binding.

**Conclusion:**

In summary, CUT4-mutant rejects whole glycosylated-nCoV2 in all pre-dock, post-dock and competitive-docking conditions. The present work strategy is relevant because it could be able to block at the first level entry of the virus to the host cells.

**Supplementary Information:**

The online version contains supplementary material available at 10.1186/s41231-022-00109-5.

## Background

COVID-19 causing coronavirus SARS-CoV-2 spreads through its spike-glycoprotein attachment to the specific amino acids of Angiotensin Converting Enzyme 2 or ACE2. The binding responsible part on the spike-glycoprotein is known as Receptor Binding Domain (RBD) [1]. The site specific blocking could be the major controlling point of this disease. Here, we have targeted the specific fraction of RBD and introduced one or more mutations in it. We noticed stronger ACE2 attachment with the mutant RBD fraction. We hypothesized that, a dose dependent application of our proposed peptide might prevent the attachment of SARS CoV-2 with the ACE2. In earlier and also in recent periods, several Angiotensin Receptor Blockers (ARBs) exhibited some protecting effects. In addition to the antihypertensive effects, these drugs manifested some anti-inflammatory effects also. The ARBs are found to be protective in severe acute respiratory syndrome caused by the virus [2]. Moreover, in some cases ACE-inhibitors and ARBs were also found to be associated with decreased mortality [3]. In contrary, it is observed that intravenous infusions of ACE inhibitors and ARBs in experimental animal model increased the expression of ACE2 receptors in the cardiopulmonary circulation. Report revealed that patients taking these therapies might be at higher risk of more viral internalization and severe disease outcomes [4]. The Renin–Angiotensin–Aldosterone-System (RAAS) blocker and statin were found to have some cardiovascular benefits but whether ACE2 blockade is effective in COVID-19 is not clear [5]. Our current study explains the efficiency of the short fraction of spike protein and its mutant fragment in blocking the ACE2 to inhibit SARS CoV-2 binding to it. One study demonstrated the bioenergetics pattern of binding-interface between the spike-glycoprotein and the ACE2 receptor. This study also tested several inhibitor peptides in SARS-CoV-2 infection [6]. The antibiotic dalbavancin was shown to bind to human ACE2 and blocked the SARS-CoV-2 spike protein [7]. This antibacterial drug may have some side effects on liver and kidney.

In this background, the present study was intended to propose a suitable and universal blocker by competing with the spike protein of the SARS CoV-2 virus. We selected the RBD sequences and structures after making an extensive comparison of the spike proteins from 186 countries. Selected amino acid T500 from the proposed RBD fraction was mutated and found that the conserved fraction CUT4 (from CUT 1, 2, 3 and 4) with T500S, Y489S, T500S and Y489S,Y453S,T500Y mutations have higher binding than the corresponding wild type fraction or the whole spike protein. This work has great therapeutic implications to develop an efficient and universal blocker of the SARS CoV-2.

## Materials and method

### Structure retrieval, analysis and prediction

The X-ray crystallographic structure of ACE2 in a bonded state with nCOV2 (PDB ID: 6VW1) [8] was retrieved from RCSB PDB [9]. It was presented at 2.68 Å resolutions and observed with an R-value of 0.199 (PDB https://doi.org/10.2210/pdb6VW1/pdb). All the amino acids involved in the binding of nCOV2 spike-glycoprotein and ACE2 was analyzed using PyMol [10]. Considering this interaction pattern as standard, other short segments were analyzed for best interaction with ACE2. The short segments were analyzed in two ways; different CUTs were prepared from tertiary structure of 6VW1 and the sequences of respective CUTs were subjected to the investigation by Swiss model Expasy server [11] for tertiary structure prediction.

### Docking studies

Different CUTs and their predicted structures were individually docked with ACE2. These Protein–Protein docking studies were performed using the Haddock [12] and Patch dock software [13] to check the binding affinity of probable structures obtained from the main one and those predicted by Swiss model Expasy. The results were further analyzed using the Pymol [10]. The docked structures were re-docked with the nCov2 to check the decrease in biding affinity of nCov2 to the ACE2. Based on the outcome of complete rejection of nCOV2 spike-protein from the CUT-docked ACE2, higher binding-affinity and smaller length of H-bond, the best CUT was selected for further analysis.

### Mutation induction and analysis

Extensive structural study was performed to understand whether the introduced mutations could elevate the binding affinity and stability (with the ACE2) of the CUT or not. Mutation sites were finalized comparing the actual interaction pattern of nCOV2 with ACE2. To optimize the process of complete displacement/ rejection of the spike-protein (from the ACE2), some point mutations, double mutations and triple mutations were introduced and there bound/ unbound structures were evaluated using the Swiss Model Expasy [11].

The studies on blind-docking of different mutated CUTs with the ACE2 were performed using the Haddock 2.4 [12] and Hawkdock [13, 14]. To obtain the best mutated structure ensuring complete nCOV2 displacement, re-docking of nCOV2 with ACE2 (pre-docked with different mutated CUT) was performed using the Haddock [12] and Hawkdock software [14]. Competitive docking was performed to analyze the binding of nCOV2 and the selected mutant with the ACE2. Further competitive docking was performed using ACE2 (PDB ID- 6VW1), the complete spike (PDB ID- 6VYB) and different selected mutated CUTs. The PRODIGY [15] was used to analyze the binding affinity/stability of the competitive docking. The best mutated structures from the docking result were accepted.

### Worldwide mutation analysis

The 186 nucleotide sequences (most dominant in the respective countries) of SARS-CoV-2 genomes isolated from humans were used in the present study. The preliminary data were collected from The Global Initiative on Sharing Avian Influenza Data (https://www.gisaid.org/) and SARS-CoV-2 spike glycoprotein gene sequence which was isolated from WUHAN –Hu-1 (COVID-19). The later was retrieved from the National Center of Biotechnology Information (NCBI) Biological database (https://www.ncbi.nlm.nih.gov/). Comparison of the selected nucleotide sequences of SARS-CoV-2 were conducted through multiple sequence alignment using CLUSTAL X2 [16]. Then spike glycoprotein gene sequences were CUT using CLC sequence viewer. These gene sequences were unique from SARS-CoV-2 spike glycoprotein gene sequence isolated from WUHAN –Hu-1. Conversion of the gene sequences into protein sequence was done by using sequence manipulation suite tool (SMS). The structure of the world wide spike protein sequences were predicted using Swiss model Expasy [11] and the structural alignment and stability analysis was done using PyMol [10].

### Analysis of the ACE1 and ACE2 receptor binding domains

The nCOV2 spike glycoprotein interacts with ACE2 receptor binding domain, but not with ACE1, though two structures are highly similar and super-imposable [17]. This comparison was performed at secondary and tertiary structure level using the server of Protein Contacts Atlas (https://www.mrc-lmb.cam.ac.uk/rajini/index.html). This is a non-covalent contact-based secondary and tertiary structure visualization and analysis server.

## Results and discussion

### Spike glycoprotein—ACE2 attachment site analysis

Attachment site analysis was performed using the X-ray crystallographic structure of ACE2; 6VW1. This PDB structure was an X-ray diffracted structure with 2.68 Å where an ACE2 interaction with spike glycoprotein was present. According to this structural arrangement, the open state of S1 protein domain of spike-glycoprotein interacts with the help of amino acids ALA475, ASN487, TYR489, GLN493, TYR453, TYR449, TYR505, GLY496, GLY502, THR500 and ASN501. These amino acids interacted with the ACE2 surface with the amino acids SER19, GLN24, TYR83, LYS31, GLU35, HIS34, ASP38, GLU37, LYS353 and TYR41. This binding was stabilized by several H-bonding ranged from 2.639–3.576 Å length.

### Peptide screening for competitive inhibition of Spike glycoprotein-ACE2 attachment

**Fig. 1:**
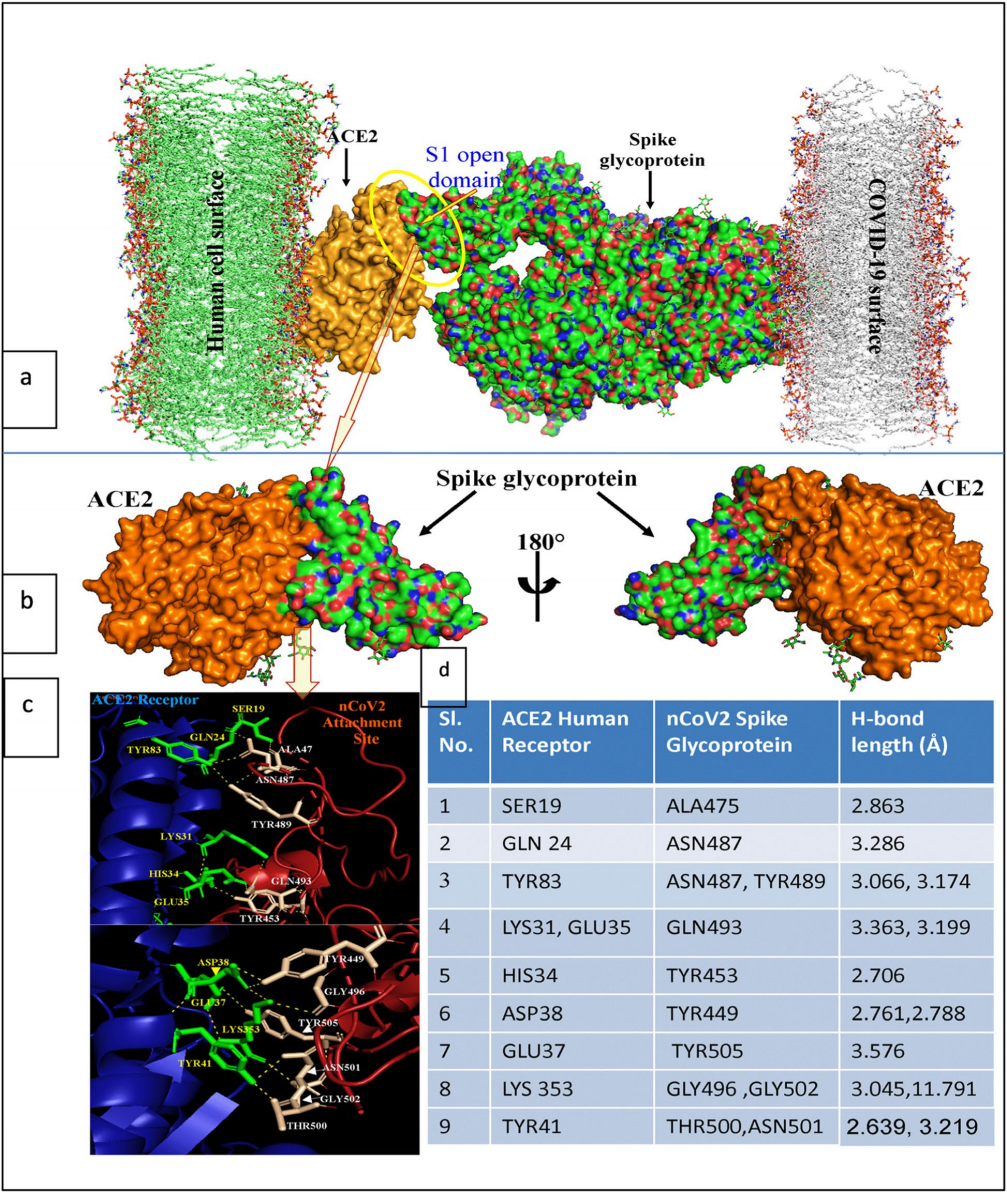
Attachment of SARS COV-2 spike glycoprotein with human ACE2 receptor. Binding interface represented in Fig **a** and **b**. Amino acid interaction pattern represented in figure **c**. Corresponding hydrogen bond length at each interaction was represented in figure **d**

To protect the entry of the SARS CoV-2 into the host cells, one strategy was taken where the blocker peptide competitively inhibits the Spike glycoprotein-ACE2 attachment. A small segment of the active site of the spike-glycoprotein was administrated for competitive inhibition study. To get effective peptide sequence as well as structure, different CUTs of spike glycoprotein and their corresponding predicted 3D structure was analyzed for the analysis of more preferable H-bonding pattern in comparison with the actual interaction as shown in Fig. 1.

**Fig. 2:**
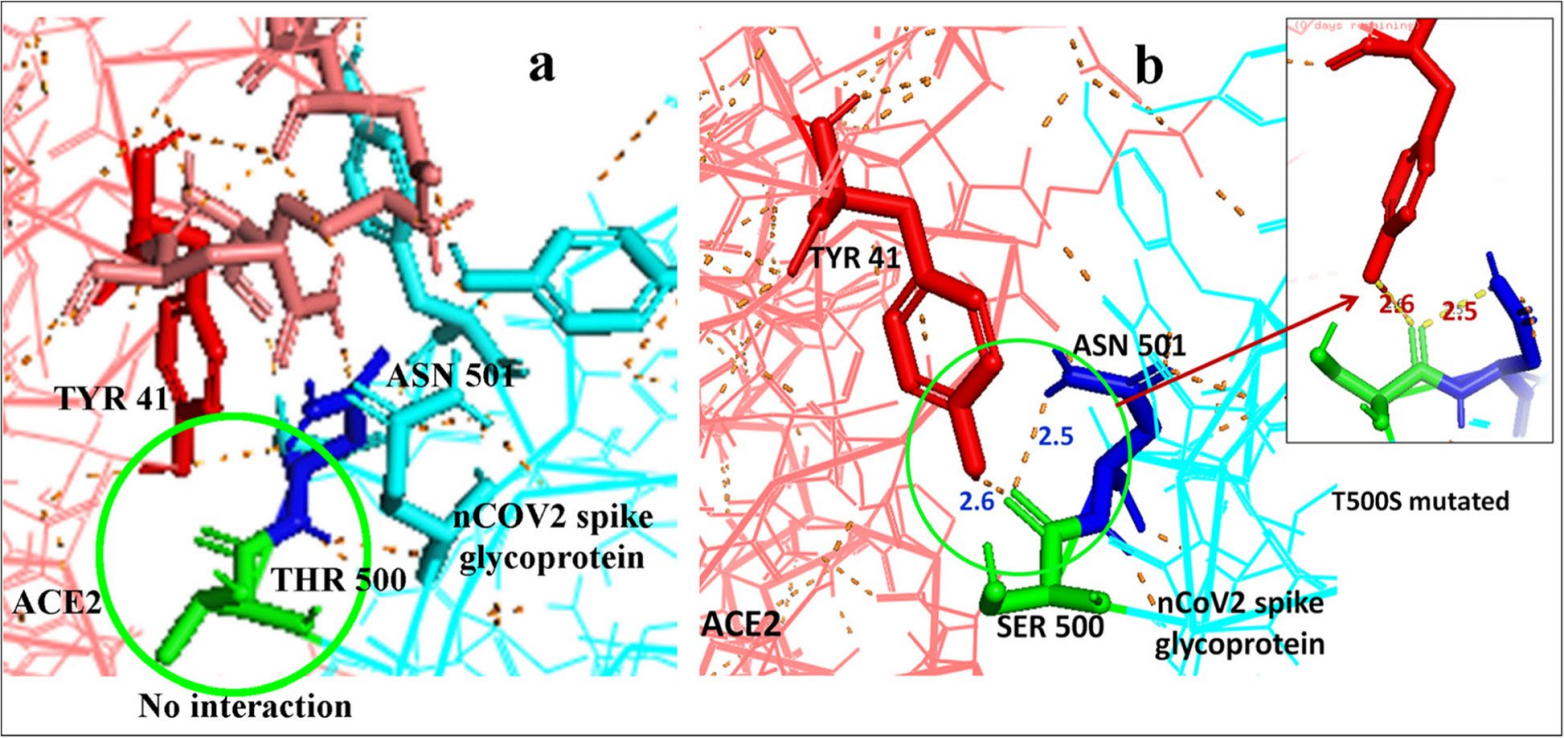
Due to H-bond based close proximity of CUT4 with ACE2 (S Table 1), unmutated CUT4-THR500 did not showed and hydrogen bond with ACE2-TYR41 (**a**). T500S mutation only replaced the larger structure of THR with SER but mode it convenient to form H-bond with ACE2-TYR41 due to presence of same –OH group (**b**) and stabilized the ACE2-CUT4 mutant binding. Finally confirms the displacement of nCOV2 spike glycoprotein binding

Initially, a peptide segment of 103 amino acids was CUT from the conserved RBD of 6VW1 spike glycoprotein and named as CUT1 (S Fig. 1). To verify the refolding capacity of CUT1, 3D structure of it was modelled using SWISS Model (CUT1S) and compared with CUT1 structure. No such structural distortion was observed between them. Then the two segments were individually subjected to molecular docking with ACE2 from 6VW1. According to the H-bonding pattern, CUT1 showed 7 interactions with ACE2 active site ranged from 1.800- 3.416 Å (S Table 1). Whereas, CUT1S showed 6 H-bond interactions ranged from 1.7- 2.7 Å. Though the affinity of CUT1 and CUT1S towards ACE2 were comparatively higher than actual (H-bond ranges from 2.639–3.576 Å), interactions of HIS34-TYR453, TYR41-THR500 were absent. And an extra H-bonding GLN42-TYR449 was present in CUT1. Whereas, in CUT1S, HIS34-TYR453, ASP38-TYR449, GLU37-TYR505 and TYR41-THR500 were absent and an extra GLN42-TYR449 was present. For competitive inhibition study the CUT1-ACE2 and CUT1S-ACE2 docked structures were individually subjected to protein–protein docking with actual spike glycoprotein from 6VW1 (S Fig. 2). Partial interactive distortion of spike glycoprotein was observed except position THR500. To get complete interactive distortion other different CUTs were similarly analyzed.

Structural comparison of CUT2 and CUT2S with 70 amino acids showed significant structural distortion with a loss of refolding capacity. The CUT2 did not showed HIS34-TYR453, GLU37-TYR505 and TYR41-ASN501 interactions and showed some extra bonds like GLN42-GLN48, LYS31-PHE490. But, an imbalance was observed between CUT2 and CUT2S interactions with ACE2 (S Table 1). No interaction was observed between CUT2S and ACE2. Multi docking of ACE2 with CUT2 and spike glycoprotein did not showed significant interaction distortion and hence it was rejected for further study.

For CUT3, 4 amino acids were extended at the C-terminal end of CUT2 and that 74 amino acid long peptide was analyzed in a similar way. Complete structural similarity was observed between CUT3 and CUT3S (S Fig. 1). But at the interaction level, common interactions of SER19-ALA475, HIS34-TYR453 and GLU37-TYR505 were absent in both. Whereas, the important interaction TYR41-ASN501, THR500 was absent in CUT3S (S Table 1). Better result was observed in multi docking attempt among CUT3-Spike-ACE2 and CUT3S-Spike-ACE2. Complete distortion of spike was observed while using CUT3. For CUT3S, spike interaction at the nearby location was observed except the active site (S Fig. 2). Though CUT3 showed better docking result but to optimize the process further peptide screening was performed.

CUT3 was extended at both C & N terminal end and termed as CUT4 with 84 amino acids and selected for the further study. The CUT4S also showed proper refolding and structural similarities with CUT4 (S Fig. 1). According to the CUT4 and CUT4S docking analysis with ACE2, 9 and 11 interaction sites, respectively were revealed. Among them 7 and 8 interactions were common for CUT4 and CUT4S, respectively in comparison with the whole spike glycoprotein. The CUT4 showed extra LYS31-PHE490 and GLN42-GLN498 interactions. In addition to that, CUT4S showed TYR41-ASN501 and GLN325-ARG439 interactions. Multi docking experiment with CUT4-ACE2-Spike showed interaction with spike protein whereas CUT4S-ACE2-spike doesn’t show any interaction with the spike glycoprotein at the specific site of attachment in comparison to CUT4.The interaction of TYR 41-ASN501 and GLN 325-ARG439 in CUT4S-ACE2 (S Table 1) for blocking the site for the attachment of Spike glycoprotein as compared to CUT4(S Fig. 1). According to the analyses of different scores generated during the Molecular Docking study with HADDOCK, the best HADDOCK score of -121.9 ± 5.6 was observed for CUT4S. That also showed the Van-der-Waals energy and Electrostatic energy value of -74.2 ± 1.0 and -167.6 ± 4.4 to be the most favourable (S Table 2). Therefore, CUT4S was taken for the mutation study and the site of interactions of several amino acids in the CUT4S-ACE2 structure were selected for this purpose. The aim was to develop some suitable mutants having better efficacies in binding/blocking the ACE2. That would prevent the interaction with spike glycoprotein.

### Mutation induction and competitive inhibition of Spike glycoprotein—ACE2 attachment

**Fig. 3:**
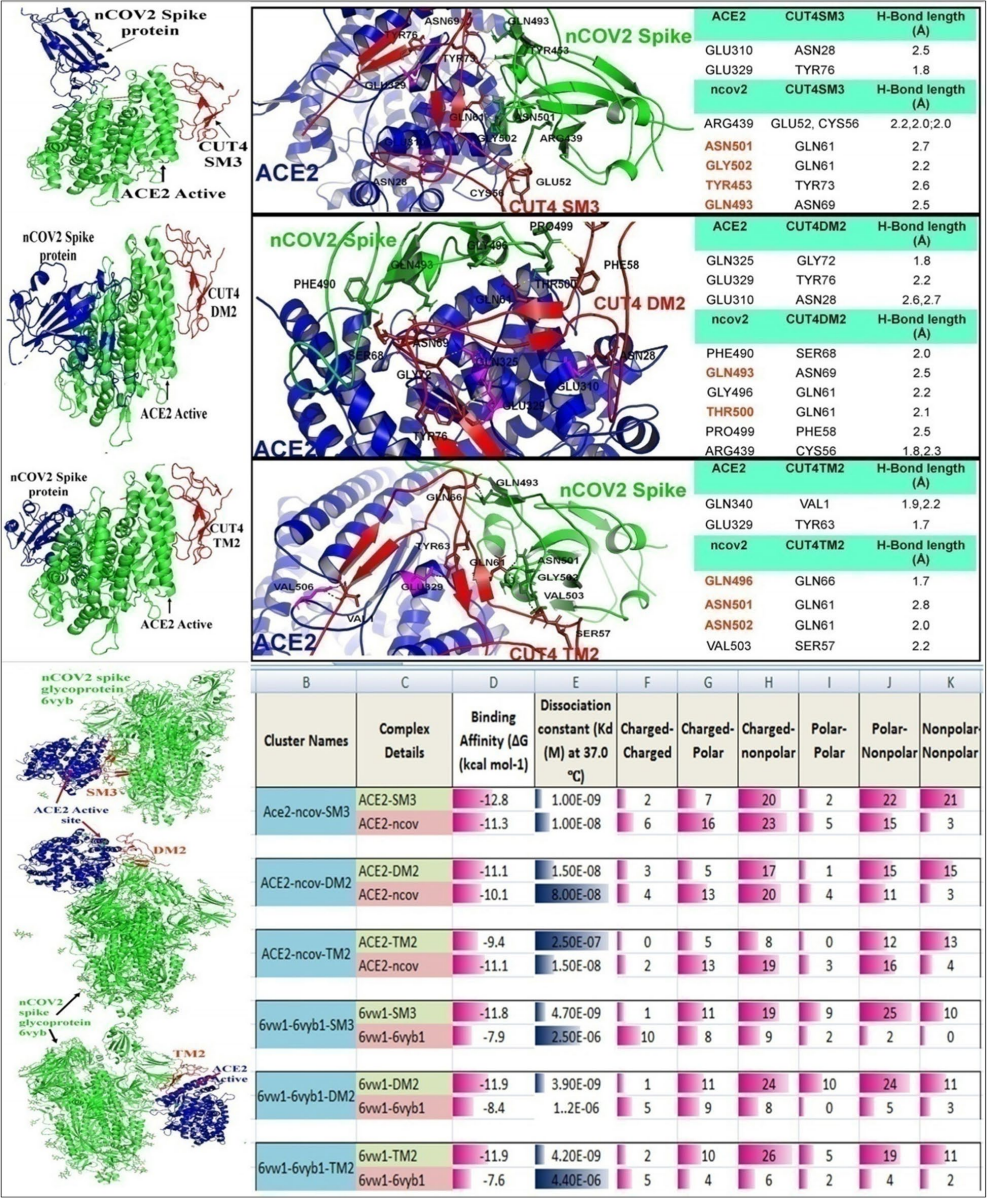
Competitive interaction of nCOV2 spike glycoprotein and Different selected mutatedCUTs with ACE2. The selected mutated CUT4s showed highest affinity to ACE2 and that attachment completely distorted the nCOV2 spike glycoprotein from ACE2 attachment (**a**, **c**, **e** and **g**). According to attachment site analysis, mutated CUT4 interacted with different active site molecules on nCOV2 spike glycoprotein, indicating their distortion with ACE2 (**b**, **d** and **f**). Figure h indicating the binding affinity of ACE2 with nCOV2 individually in presence of SM3, DM2 and TM2. Where, SM3 and DM2 showed more affinity to ACE2. Whereas for complete spike glycoprotein (6vyb1), Mutated CUT4 get more preference in ACE2 attachment as they showed binding affinity of -11.8, -11.9 and -11.9 respectively. And all SM3, DM2 and TM2 showed less dissociation from ACE2 in comparison to nCOV2 spike

The mutations were induced at the positions of TYR489, TYR453 and THR500. These three amino acids of main nCOV2 spike glycoprotein showed interactions with TYR83, HIS34 and TYR41, respectively at the ACE2 surface (Fig. 1). The amino acid threonine (THR) possess a hydroxyl (-OH) group at its R group. At location THR500, that –OH group forms hydrogen bond with the R group of TYR41 of ACE2. As Serine (SER) and Tyrosine (TYR) also have R specific hydroxyl (-OH) group, substitution with SER and TYR were made at the selected locations. The overall hydrogen bond length of CUT4 with ACE2 was less than the actual spike interaction (S Table 1, Fig. 1) and when SER was introduced at the positions of 489 and 453 to avoid the steric hindrance due to the large R group of TYR binding effect was noticed to be better. And both SER and TYR were separately introduced at the location of THR500 to verify their impact. Different unmutated CUTs and mutated CUT4 combination of single mutation (4 structures), double mutations (5 structures) and triple mutations (2 structures) were analyzed (S Fig. 3). In total 11 structures were predicted using Swiss Model and individually docked with ACE2. In single mutation Y489S, Y453S and T500Y did not show proper interactions (S Table 3) and hence were rejected for further study. Whereas, T500S mutation showed hydrogen bonding with TYR41 of ACE2, which mimicked the actual ACE2-nCOV2 spike-binding features (Fig. 2). This interaction was initially lost in case of CUT 4 and hence SM3 was considered for further study. Double Mutation 1(DM1) with Y489S Y453S showed no interaction at the mutated site and comparatively at lesser interacting sites as compared to SM3 hence, those were rejected for further study. DM2 with Y489S T500S showed same number of interacting sites as compared to SM3. Here mutated SER 500 bonded with TYR41 with a bond length of 1.8 Å hence was also considered for further study. DM3 with Y489S T500Y, DM4 with Y489S T500Y and DM5 with Y453S T500Y, all showed fewer interacting sites as compared to DM2 (S Table 3) with no interaction at the mutated sites (S Fig. 2) and hence were all rejected for further studies. Triple Mutation 1(TM1) with Y489S Y453S T500S mutation also showed no interaction at the mutation site hence not considered for further study. TM2 with Y489S, Y453S, T500Y though had same number of interacting sites as TM1 (S. Table 3) showed interaction of TYR500 with that of TYR41 with Hydrogen bond length 2.6 Å(S Fig. 2). Hence the selected three sets were considered for further studies. Finally, three combination of mutation SM3, DM2 and TM2 individually showed mimicked interaction with ACE2 in competition with nCOV2 spike protein.

The docked structure ACE2-CUT4 SM3, ACE2-CUT4 DM2 and ACE2-CUT4 TM2 were further used for multiple docking with nCOV2 spike protein (Fig. 3). The nCOV2 spike protein showed partial interaction with ACE2 active site in presence of CUT4 main, but completely detached from ACE2 active site in presence of CUT4S. This uncertainty and incompetence in the blocking effect was further resolved by introducing some specific mutations at above mentioned position. For all the selected mutations SM3, DM2 and TM2, nCOV2 spike protein showed complete displacement/rejection from ACE2 active site. Which indicated that, pre-administration of these selected mutated peptides in physiological condition, would inhibit the nCOV2 spike protein interaction with ACE2 (Fig. 3 a, c and e).

To understand the competitive interaction of nCOV2 spike protein and individual mutated peptideCUT4 SM3, CUT4 DM2 and CUT4 TM2 with ACE2, a multi ligand docking study was performed with ACE2 using the HADDOCK. Results represented that the ACE2 is partially docked by both of nCOV2 spike and CUT4 mutants at the active site. In addition to that, some of the nCOV2 spike proteins active site amino acids were blocked by CUT4 mutants (Fig. 3b). For single mutation, ASN501, GLY502, TYR453 and GLN493 of nCOV2 were blocked by CUT4SM3. These amino acids stabilize the interaction with ACE2 at the middle and lower position. Similarly CUT4DM2 and CUT4TM2 were also showed significant interactions with nCOV2 spike active site (Fig. 3d and f). This competitive interaction results that if sufficient quantities of CUT4 mutants are available at the physiological system, they would destabilize the ACE2-nCOV2 spike interaction. For further confirmation, a complete glycosylated nCOV2 spike protein was selected for ACE2 interaction study in presence of CUT4 SM3, CUT4 DM2 and CUT4 TM2 individually. Results indicated the complete ACE2-nCOV2 inhibition in all cases as shown in Fig. 3g. The competitive interaction study was further analyzed through Binding affinity (Fig. 3h). Figure h indicating the binding affinity of ACE2 with nOCV2 individually in presence of SM3, DM2 and TM2. Where, SM3 and DM2 showed binding affinity of -12.8 and -11.1 in comparison with ACE2-nCOV2 interaction with the binding affinity value of -11.3 and -10.1 respectively. On the other hand the complete spike glycoprotein binding affinity was low or moderate comparative to ACE2-CUT4 mutant binding. This result indicated that different CUT4 mutant might be able to inhibit ACE2-nCOV2 spike protein interaction.

**Fig. 4:**
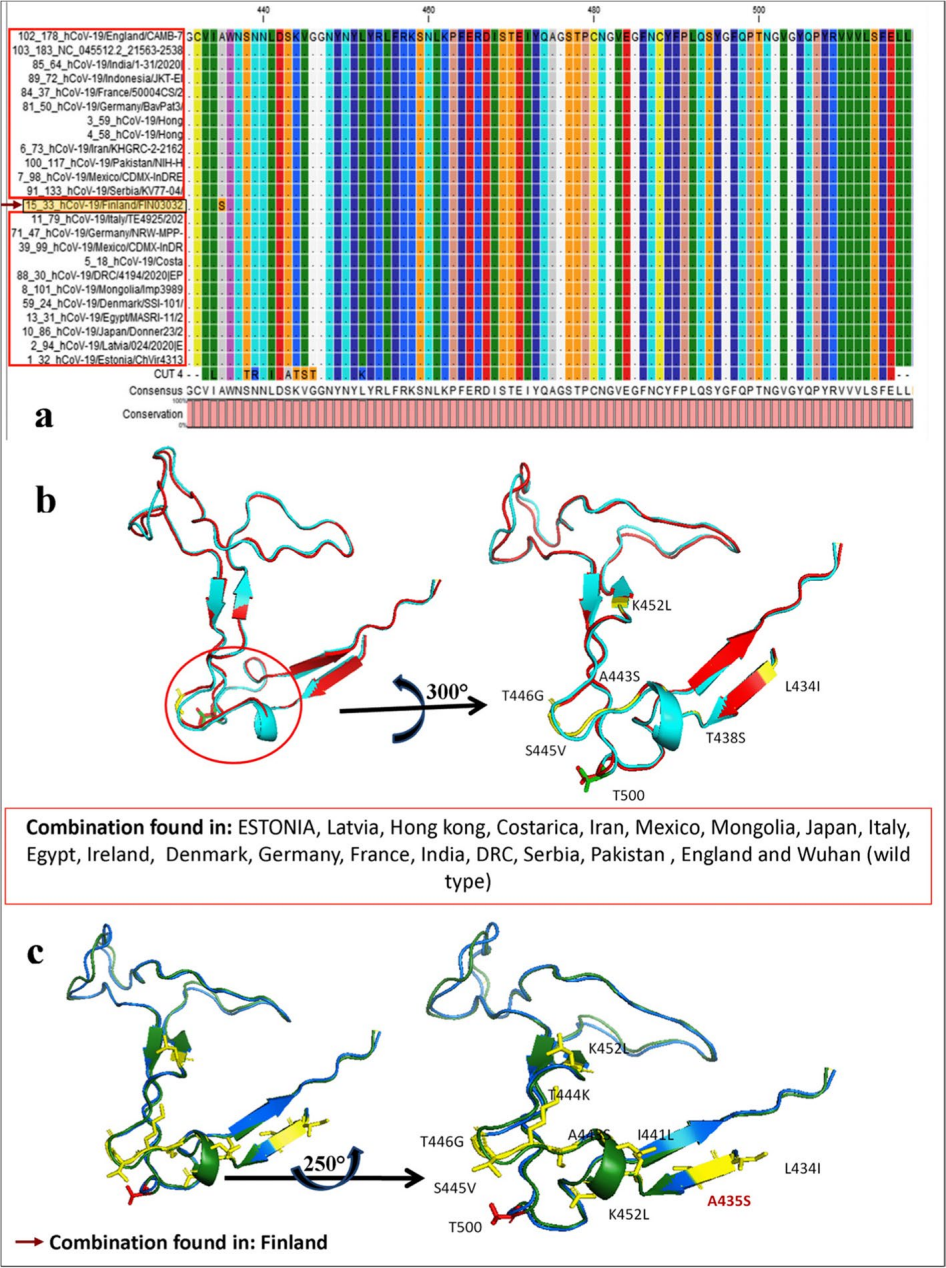
CUT4 sequence alignment with 24 unique spike proteins from different countries. These 24 unique sequences were selected from 186 spike protein sequences representing 105 countries (**a**). Sequence diversity was observed in sequence FIN30302 where one Ser was observed at alignment position 435 and in CUT4 within alignment position 452. Whereas, all other sequences showed identical sequences over the CUT regions. The sequence diversity with CUT4 did not reflected at the T500 position for both **b** and **c**. Which indicated that mutated CUT4 can able to compete with all type of COVID -19 spike glycol proteins throughout 105 countries in the world

Finally, the presence of CUT4 was studied among 186 COVID-19 spike proteins from 105 different countries. Those sequences were subjected to common sequence shorting. The result indicated 24 unique sequences among all (Fig. 4a). According to the alignment study, one sequence from Finland showed a substitution of S instead of A. But this change has not reflected in T500 position upon which the complete displacement of nCOV2 spike is dependent. Other common conserved pattern from different countries like; ESTONIA, Latvia, Hong Kong, Costarica, Iran, Mexico, Mongolia, Japan, Italy, Egypt, Ireland, Denmark, Germany, France, India, DRC, Serbia, Pakistan, England and Wuhan (wild type)also depicted the conserved T500 (Fig. 4b and c). It is indicating the universality of our CUT4 mutants.

**Fig. 5:**
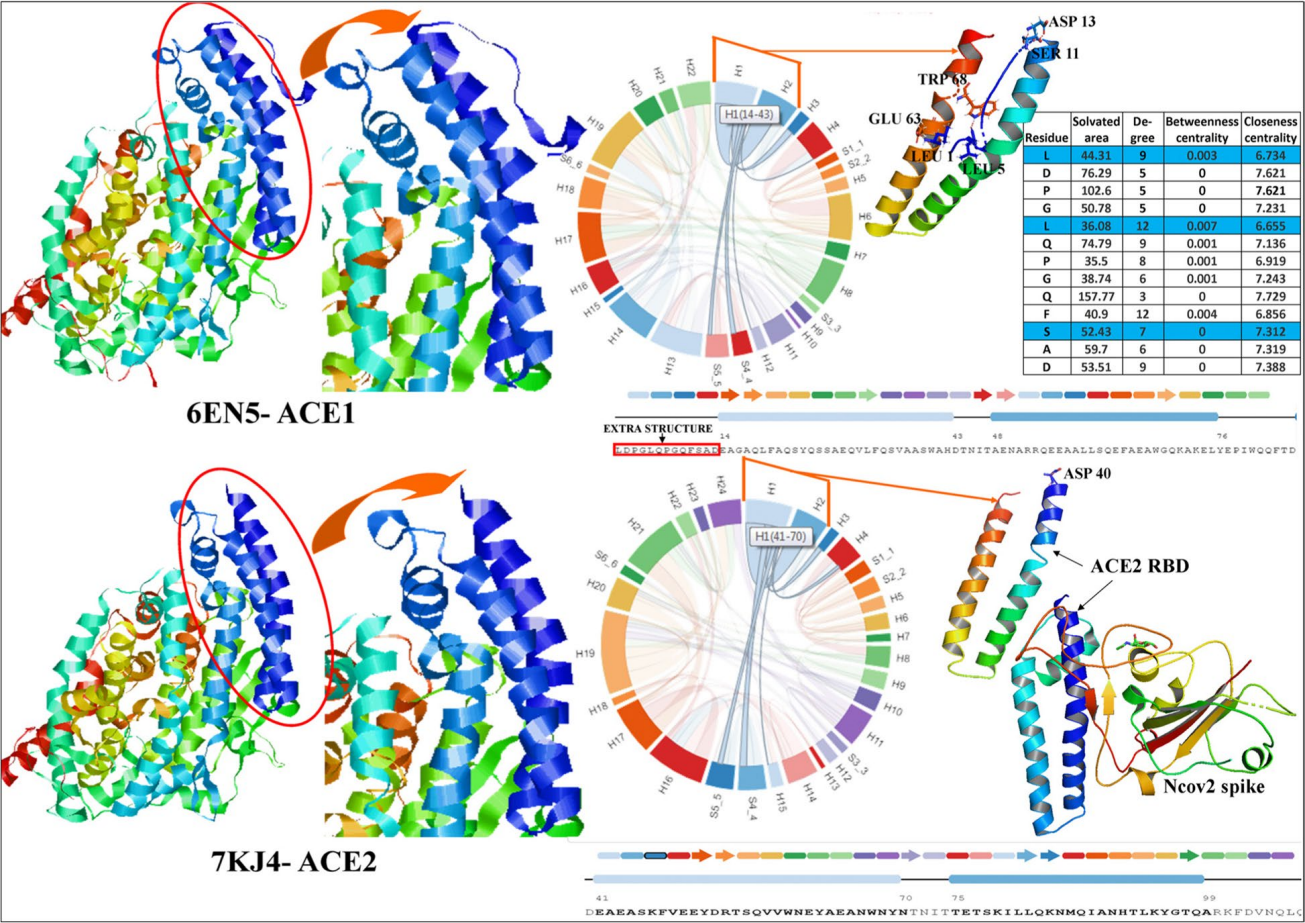
Comparative study between ACE1 and ACE2 receptor binding domain (RBD). Though they have sequence, secondary and tertiary structure level similarity, ACE1 have extra appendage which interacted with the RBD domain with amino acids combination of LEU1-GLU63, LEU5- TRP68 and SER11- ASP13, which blocks the nCOV spike attachment with ACE1. Whereas, ACE2 has no extra part which facilitated the nCOV2 spike binding

According to the literature, ACE2 is the sole receptor of SARS-CoV-2 spike glycoprotein but not ACE1, though two structures are similar. With reference to that, we have studied the RBD of ACE1 and ACE2 (Fig. 5). Two structures were similar at sequence, secondary and tertiary level also. Only, the Helix1 (H1) of ACE1 started from residue 14 but H1 of ACE2, started from residue 41.ACE2 has an extra appendage at the N terminal start with the amino acid composition of (1)LDPGLQPGQFSAD(13). Where, LEU1, LEU5, SER11 interacted with the GLU63, TRP68 and ASP13. This interaction blocked the RBD domain of ACE1 as compared to ACE2. Quantitative values like solvated area, degree, betweenness centrality and closeness centrality of LEU1, LEU5, SER11 interaction has also been presented in Fig. 5, which indicated that, structural involvement of this extra appendage with RBD domain. The RBD domain of ACE2 remain uncovered as according to the PDB structures 1r4l, 1r42, 3d0h, 3d0i, 3scj, 3sck, 3scl, 7kj3 and 7kj4. Whereas structure of ACE1 (6en5) showed the four unit with that extra appendage. So, nCOV2 spike attachment to ACE2 has been facilitated due to the hassle less interaction in comparison to ACE1.

According to Ali and Vijayan (2020) [17], the mechanism of nCOV2-ACE2 interaction starts near the N-terminus of ACE2 with the amino acids ofAsp38, Glu35 and Lys353 which has also decided in Fig. 1. So, in our study, selection of CUT segment throughout the RBD domain is significant rather than a partial one. This will confirm the complete detachment of nCoV spike binding. Report revealed that synthetic inhibitor comprising two α helical peptides homologues to protease domain of ACE2 can block the RBD domain of S protein of the SARS CoV 2. This significant docking may have some therapeutic implications also. Similar type of study suggests that engineered ACE2 receptor traps can neutralize SARS-CoV-2[18]**.** A hexa-peptide in core of spike RBD inhibits the association between spike S1 and ACE-2 [19].

This work has been shown in cultured human cells and in experimental animal model. But as this peptide is a very short fragment having no higher-order of protein structure so the effect may not be due to the complementarily based S1 and ACE-2 binding. Several organ-protecting effects of this hexapeptide justify more intense work in this aspect [19]. However, in the current study we demonstrated that in-silico specific amino acid alteration in the highly conserved S protein of the SARS CoV 2 can potentially block its ACE2 binding, hence minimize the viral infection.

## Conclusions

The present study has immense therapeutic implication in COVID-19 research. The spike proteins of the SARS CoV 2 from the 186 countries of origin were compared by alignment software. Different conserved fraction and conserved amino acids were targeted for mutation to test the ACE2 blocking effects. For this purpose several CUT sequences (CUT 1–4) were verified stepwise and found that the CUT4 (84 amino acid sequences in RBD) to be most effective as a blocker. It was further noticed that single mutation- T500S, double mutations- Y489S/T500S and triple mutations- Y489S/Y453S/T500Y could be great universal ACE2 blockers. Amongst all the mutations, the single T500S mutation was found to be most effective. This work has promising therapeutic applications. Further studies are necessary.

## Supplementary Information

**Additional file 1: Table S1.** Hydrogen bond pattern of different CUTs (CUT1-CUT4). The sequences were subjected to structure prediction through SWISS model and that was used for molecular docking with ACE2. Both structure CUT and modeled structures were analyzed for H-bond length analysis where good binding pattern and short H-bond length was found for CUT4 structures. **Table S2.** The docking parameters for Cut 1 ,2,3,4 (normal and Swiss model) have been tabulated. **Table S3**. Indicates the Single, Double and Triple mutation induced Cut 4 autodock result analysis with ACE2 receptor depicting the site of interaction as well as the Hydrogen bond length in each case.T 500 S mutation showed best results.

**Additional file 2: Figure S1**. Different Cut site analysis of SARS COV-2 spike glycoprotein. **Figure S2.** Binding interaction site analysis between Cut 1,2,3,4 and ACE Receptor. **Figure S3.** Depicts the binding sites for Cut 4 Main unmutated with ACE 2 receptor and the resultant interaction in the case of Single Mutation, Double Mutation and Triple mutation induced in Cut 4 with the same.

## Abbreviations

ACE: Angiotensin-converting enzyme
ACE2: Angiotensin-converting enzyme2
ALP: Alkaline phosphatase
ARBs: Angiotensin Receptor Blockers
CASTp: Computed-Atlas-of-Surface-Topography
Covid-19: Coronavirus disease 2019
EGCG: Epigallocatechin-gallate
ELISA: Enzyme-linked immunosorbent assay
RAAS: Renin–angiotensin–aldosterone system
RBD: Receptor binding domain
SARS-CoV-2: Severe acute respiratory syndrome coronavirus 2
TNF-α: Tumor necrosis factor-α
WHO: World Health Organization
