# Supplementary material for: Global conserved RBD fraction of SARS-CoV-2 S-protein with T500S mutation in silico significantly blocks ACE2 and rejects viral spike
Source: Transl Med Commun. 2022 Feb 4;7(1):2. doi: 10.1186/s41231-022-00109-5 (PMC8814807; doi:10.1186/s41231-022-00109-5)
Supplement: Supplementary file 1 — Additional file 1: Table S1. Hydrogen bond pattern of different CUTs (CUT1-CUT4). The sequences were subjected to structure prediction through SWISS model and that was used for molecular docking with ACE2. Both structure CUT and modeled structures were analyzed for H-bond length analysis where good binding pattern and short H-bond length was found for CUT4 structures. Table S2. The docking parameters for Cut 1 ,2,3,4 (normal and Swiss model) have been tabulated. Table S3. Indicates the Single, Double and Triple mutation induced Cut 4 autodock result analysis with ACE2 receptor depicting the site of interaction as well as the Hydrogen bond length in each case.T 500 S mutation showed best results. [file 41231_2022_109_MOESM1_ESM.docx]

| Sl No. | **CUT 1** | | | | | | **CUT 2** | | | **CUT 3** | | | | | | **CUT 4** | | | | | |
| --- | --- | --- | --- | --- | --- | --- | --- | --- | --- | --- | --- | --- | --- | --- | --- | --- | --- | --- | --- | --- | --- |
|  | MGCVLAWNTRNIDATSTGNYNYKYRLFRKSNLKPFERDISTEIYQAGSTPCNGVEGFNCYFPLQSYGFQPTNGVGYQPYRVVVLSFEAPATVCGPKLSTDLIK | | | | | | TRNIDATSTGNYNYKYRLFRKSNLKPFERDISTEIYQAGSTPCNGVEGFNCYFPLQSYGFQPTNGVGYQP | | | TRNIDATSTGNYNYKYRLFRKSNLKPFERDISTEIYQAGSTPCNGVEGFNCYFPLQSYGFQPTNGVGYQPYRVV | | | | | | VLAWNTRNIDATSTGNYNYKYRLFRKSNLKPFERDISTEIYQAGSTPCNGVEGFNCYFPLQSYGFQPTNGVGYQPYRVVVLSFE | | | | | |
|  |  |  |  |  |  |  |  |  |  |  |  |  |  |  |  |  |  |  |  |  |  |
|  | **Cut 1 main** | | | **Cut 1 swiss** | | | **Cut 2 main** | | | **Cut 3 main** | | | **Cut 3 Swiss** | | | **Cut 4 main** | | | **Cut 4 swiss** | | |
|  | ACE2 Human Receptor | nCoV2 Spike Glycoprotein | H-bond length | ACE2 Human Receptor | nCoV2 Spike Glycoprotein | H-bond length | ACE2 Human Receptor | nCoV2 Spike Glycoprotein | H-bond length | ACE2 Human Receptor | nCoV2 Spike Glycoprotein | H-bond length | ACE2 Human Receptor | nCoV2 Spike Glycoprotein | H-bond length | ACE2 Human Receptor | nCoV2 Spike Glycoprotein | H-bond length | ACE2 Human Receptor | nCoV2 Spike Glycoprotein | H-bond length |
| 1 | SER19 | ALA475 | 2.336 | SER19 | ALA475 | 1.7 | SER19 | SER 477,ALA475, | 2.8,1.8 | GLN 24 | ASN50 | 2.1 | GLN 24 | ASN50 | 2 | SER19 | ALA 43 | 1.8 | SER19 | ALA 475 | 1.8 |
| 2 | GLN 24 | ASN487 | 1.879 | GLN 24,TYR83 | ASN487 | 1.8,2 | GLN 24 | ASN487 | 2.2 | TYR83 | ASN487 | 2 | TYR83 | ASN50 | 2 | GLN 24 | ASN55 | 1.9 | GLN 24 | ASN 487 | 1.9 |
| 3 | TYR83 | ASN487 | 2.117 | LYS31, GLU35 | GLN493 | 2,1.7 | TYR83 | ASN487 | 2.2 | LYS31, GLU35 | GLN493 | 1.9,1.7 | LYS31, GLU35 | GLN56 | 1.7,1.8 | TYR83 | ASN55 | 1.9 | TYR83 | ASN 487 | 1.9 |
| 4 | LYS31, GLU35 | GLN493 | 3.416, 1.800 | GLN42 | TYR449 | 2.4 | LYS31 | PHE490 | 1.9 | ASP38, GLN42 | TYR449 | 1.6,2.2 | ASP38, GLN42 | TYR12 | 1.7,2.7 | LYS31 | PHE58 | 1.9 | LYS31 | PHE490 | 1.9 |
| 5 | ASP38, GLN42 | TYR449 | 2.477, 2.371 | LYS 353 | GLY496 ,GLY502 | 2.3,2.1 | LYS31, GLU35 | GLN493 | 1.7,1.7 | TYR41 | ASN501, THR500 | 1.9,2.4 | LYS 353 | GLY59 , GLY65 | 2.1,2.2 | LYS31, GLU35 | GLN61 | 2,1.8 | LYS31, GLU35 | GLN493 | 1.7,1.7 |
| 6 | LYS 353 | GLY496 ,GLY502 | 3.396, 2.014 | TYR41 | ASN501, | 2.7 | ASP38, | TYR449 | 1.7,2.371 | LYS 353 | GLY502, 496 | 2.6,1.9 |  | | | ASP38, | TYR17 | 1.9 | GLU37, | TYR505 | 1.7 |
| 7 | TYR41 | ASN501, | 2.288 |  | | | GLN42 | GLN48 | 2.7 |  | | | | | | GLN42 | GLN66 | 1.9 | GLN42 | GLN498 | 1.9 |
| 8 |  | | | | | | LYS 353 | GLY496 ,GLY502 | 2.5,1.9 |  |  |  |  |  |  | LYS 353 | GLY64 , GLY70 | 2.6,1.8 | LYS 353 | GLY496 ,GLY502 | 1.9,2 |
| 9 |  |  |  |  |  |  | TYR41 | THR500 | 1.7 |  |  |  |  |  |  | GLU37 | TYR73 | 1.8 | ASP38 | TYR449 | 1.8 |
| 10 |  | | | | | | | | | | | | | | | | | | TYR41 | ASN501 | 2.6 |
| 11 |  |  |  |  |  |  |  |  |  |  |  |  |  |  |  |  |  |  | GLN325 | ARG439 | 2.4 |

**S Table1.** Hydrogen bond pattern of different CUTs (CUT1-CUT4). The sequences were subjected to structure prediction through SWISS model and that was used for molecular docking with ACE2. Both structure CUT and modeled structures were analyzed for H-bond length analysis where good binding pattern and short H-bond length was found for CUT4 structures.

| **Parameters** | **CUT 1 Normal** | **CUT 1 Swissmodel** | **CUT 2 Normal** | **CUT 2 Swissmodel** | **CUT 3 Normal** | **CUT 3 Swissmodel** | **CUT 4 Normal** | **CUT 4 Swissmodel** |
| --- | --- | --- | --- | --- | --- | --- | --- | --- |
| **HADDOCK score** | -117.4 +/- 3.8 | -115.2 +/- 2.4 | -120.3 +/- 2.2 | -0.2 +/- 0.0 | -118.1 +/- 3.2 | -117.6 +/- 7.6 | -119.9 +/- 1.8 | -121.9 +/- 5.6 |
| **Cluster size** | 20 | 20 | 20 | 1 | 20 | 20 | 20 | 20 |
| **RMSD from the overall lowest-energy structure** | 0.3 +/- 0.2 | 0.3 +/- 0.2 | 0.3 +/- 0.2 | 0.0 +/- 0.0 | 0.3 +/- 0.2 | 0.3 +/- 0.2 | 0.3 +/- 0.2 | 0.3 +/- 0.1 |
| **Van der Waals energy** | -74.2 +/- 3.1 | -73.3 +/- 1.5 | -75.1 +/- 3.1 | 0.0 +/- 0.0 | -73.3 +/- 1.4 | -74.8 +/- 4.8 | -71.8 +/- 2.1 | -74.2 +/- 1.0 |
| **Electrostatic energy** | -152.4 +/- 11.7 | -141.2 +/- 28.1 | -144.9 +/- 17.0 | 0.0 +/- 0.0 | -128.1 +/- 6.7 | -142.0 +/- 30.1 | -185.1 +/- 9.7 | -167.6 +/- 4.4 |
| **Desolvation energy** | -12.7 +/- 4.2 | -13.7 +/- 6.5 | -16.2 +/- 3.0 | -0.2 +/- 0.0 | -19.1 +/- 3.6 | -14.5 +/- 12.3 | -11.1 +/- 2.3 | -14.2 +/- 5.5 |
| **Restraints violation energy** | 0.1 +/- 0.05 | 0.0 +/- 0.00 | 0.0 +/- 0.00 | 0.0 +/- 0.00 | 0.0 +/- 0.00 | 0.0 +/- 0.00 | 0.0 +/- 0.00 | 0.0 +/- 0.00 |
| **Buried Surface Area** | 1968.6 +/- 36.3 | 1956.1 +/- 25.9 | 1954.4 +/- 15.7 | -999999.0 +/- 0.0 | 1921.4 +/- 20.7 | 1933.7 +/- 33.3 | 1971.8 +/- 41.4 | 1932.2 +/- 9.5 |
| **Z-Score** | 0.0 | 0.0 | 0.0 | -1.8 | 0.0 | 0.0 | 0.0 | 0.0 |

**S Table2:** The docking parameters for Cut 1 ,2,3,4 (normal and Swiss model) have been tabulated.

.

| Sl No. | MUTATION IN CUT 4  VLAWNTRNIDATSTGNYNYKYRLFRKSNLKPFERDISTEIYQAGSTPCNGVEGFNCYFPLQSYGFQPTNGVGYQPYRVVVLSFE | | | | | | | | | | | | | | | | | | | | | | | | | | | | | | | | |
| --- | --- | --- | --- | --- | --- | --- | --- | --- | --- | --- | --- | --- | --- | --- | --- | --- | --- | --- | --- | --- | --- | --- | --- | --- | --- | --- | --- | --- | --- | --- | --- | --- | --- |
|  | SINGLE MUTATION | | | | | | | | | | | | DOUBLE MUTATION | | | | | | | | | | | | | | | TRIPLE MUTATION | | | | | |
|  | MUTATION 1-Y489S | | | MUTATION 2-Y453S | | | MUTATION 3-T500S | | | MUTATION 4-T500Y | | | MUTATION 1-Y489S Y453S | | | MUTATION 2-Y489S T500S | | | MUTATION 3-Y489S T500Y | | | MUTATION 4-Y453S T500S | | | MUTATION 5-Y453S T500Y | | | MUTATION 1-Y489S Y453S T500S | | | MUTATION 2-Y489S Y453S T500Y | | |
|  | ACE2 | nCoV2 | H-BL | ACE2 | nCoV2 | H-BL | ACE2 | nCoV2 | H-BL | ACE2 | nCoV2 | H-BL | ACE2 | nCoV2 | H-BL | ACE2 | nCoV2 | H-BL | ACE2 | nCoV2 | H-BL | ACE2 | nCoV2 | H-BL | ACE2 | nCoV2 | H-BL | ACE2 | nCoV2 | H-BL | ACE2 | nCoV2 | H-BL |
| 1 | SER19 | ALA 475 | 1.7 | GLN 24 | ASN 487 | 1.8 | SER19 | ALA 475 | 1.8 | GLN 24 | ASN 487 | 1.8 | SER19 | ALA 475 | 1.7 | SER19 | ALA 475 | 1.9 | SER19 | ALA 475 | 1.7 | SER19 | ALA 475 | 1.8 | GLN 24 | ASN 487 | 1.8 | SER19 | ALA 475 | 1.7 | SER19 | ALA 475 | 1.7 |
| 2 | GLN 24 | ASN 487 | 1.9 | TYR83 | ASN 487 | 2.1 | GLN 24 | ASN 487 | 1.9 | TYR83 | ASN 487 | 2.2 | GLN 24 | ASN 487 | 1.9 | GLN 24 | ASN 487 | 1.9 | GLN 24 | ASN 487 | 1.9 | GLN 24 | ASN 487 | 1.8 | TYR83 | ASN 487 | 1.8 | GLN 24 | ASN 487 | 2.0 | GLN 24 | ASN 487 | 1.9 |
| 3 | TYR83 | ASN 487 | 2.2 | LYS31 | PHE 490,LEU60 | 2.1,2.6 | TYR83 | ASN 487 | 2.0 | LYS31 | PHE 490 | 2.1 | TYR83 | ASN 487 | 2.2 | TYR83 | ASN 487 | 1.9 | TYR83 | ASN 487 | 2.2 | TYR83 | ASN 487 | 2.2 | LYS31 | LEU60,PHE 490 | 2.7,1.8 | TYR83 | ASN 487 | 2.3 | TYR83 | ASN 487 | 2.1 |
| 4 | LYS31 | PHE 490 | 1.8 | LYS31, GLU35 | GLN493 | 1.8,1.8 | LYS31 | PHE 490 | 1.9 | LYS31, GLU35 | GLN493 | 1.9,1.8 | LYS31 | LEU492 | 2.8 | LYS31 | PHE 490 | 2.2 | LYS31 | LEU492,PHE 490 | 2.4,2.1 | LYS31 | PHE 490 | 2.1 | LYS31, GLU35 | GLN493 | 1.8,1.8 | LYS31 | PHE 490 | 2.1 | LYS31 | LEU490,PHE 490 | 2.8,2.2 |
| 5 | LYS31, GLU35 | GLN493 | 1.9,1.8 | GLN42 | TYR449 | 1.9 | LYS31, GLU35 | GLN493 | 2.9,1.7 | GLU37, | TYR505 | 2.8 | LYS31, GLU35 | GLN493 | 2.5, | LYS31, GLU35 | GLN493 | 2.1,1.8 | LYS31, GLU35 | GLN493 | 1.8,1.8 | LYS31, GLU35 | GLN493 | 1.7,1.8 | GLU37, | TYR505 | 2.3 | LYS31, GLU35 | GLN493 | 1.7,1.7 | LYS31, GLU35 | GLN493 | 1.8,1.7 |
| 6 | GLN42 | GLN 498 | 1.9 | LYS 353 | GLN496 ,GLY502 | 1.9,2.1 | GLU37, | TYR73 | 1.8 | GLN42 | GLN 498,TYR449 | 2.2,1.8 | GLN42 | GLN 498, | 1.9 | GLN42 | GLN 498, | 2.4 | GLN42 | GLN 498, | 1.9 | LYS 353 | GLN 496 ,GLY502 | 1.9,2.4 | GLN42 | GLN 498,TYR449 | 2.0, | GLU37, | TYR505 | 1.7 | GLN42 | GLN 498 | 2.2 |
| 7 | LYS 353 | GLN 496 ,GLY502 | 1.9,2.1 | ASP38 | TYR449 | 1.8 | GLN42 | GLN 498 | 1.9 | LYS 353 | GLN 496 ,GLY502 | 1.9,2.0 | LYS 353 | GLY502 | ,2.1 | LYS 353 | GLN 496 ,GLY502 | 1.9,2.1 | LYS 353 | GLN 496 ,ASN501, GLY502 | 1.9,2.6,1.9 | HIS34 | TYR495 | 3.1 | LYS 353 | GLN 496 ,GLY502 | 2.4,2.2 | GLN42 | TYR449 | 1.8 | LYS 353 | GLN 496 ,GLY502 | 2.2,1.8 |
| 8 | ASP38 | TYR449,GLN 498 | 1.7,1.9 | GLN325 | ARG439 | 1.8 | LYS 353 | GLN 496 ,GLY502 | 1.9,2.0 | ASP38 | TYR449 | 1.7 | TYR41 | THR500 | 1.7 | ASP38 | TYR449 | ,1.7 | TYR41 | ASN501 | 2.3 |  | | | ASP38 | GLN 498 | 2.0, | LYS 353 | GLN 496 ,GLY502 | 2.2,2.1 | ASP38 | TYR449,GLN 498 | 1.7,2.2 |
| 9 | TYR41 | THR500 | 1.8 |  | | | ASP38 | TYR449 | 1.8 |  | | | ASP38 | GLN 498,TYR449 | 2.3,1.7 | GLN325 | ARG439 | 2.5 | ASP38 | GLN 498,TYR449 | 1.9,1.8 |  | | | | | | ASP38 | TYR449 | 1.8 | TYR41 | TYR500 | 2.6 |
| 10 |  | | | | | | TYR41 | SER500 | 2.0 |  | | | | | | TYR41 | SER500 | 1.8 |  | | | | | | | | | | | | | | |
|  | | | | | | | | | | | | | | | | | | | | | | | | | | | | | | | | | |

**S Table3**. Indicates the Single, Double and Triple mutation induced Cut 4 autodock result analysis with ACE2 receptor depicting the site of interaction as well as the Hydrogen bond length in each case.T 500 S mutation showed best results.
