# Supplementary material for: Global conserved RBD fraction of SARS-CoV-2 S-protein with T500S mutation in silico significantly blocks ACE2 and rejects viral spike
Source: Transl Med Commun. 2022 Feb 4;7(1):2. doi: 10.1186/s41231-022-00109-5 (PMC8814807; doi:10.1186/s41231-022-00109-5)
Supplement: Supplementary file 2 — Additional file 2: Figure S1. Different Cut site analysis of SARS COV-2 spike glycoprotein. Figure S2. Binding interaction site analysis between Cut 1,2,3,4 and ACE Receptor. Figure S3. Depicts the binding sites for Cut 4 Main unmutated with ACE 2 receptor and the resultant interaction in the case of Single Mutation, Double Mutation and Triple mutation induced in Cut 4 with the same. [file 41231_2022_109_MOESM2_ESM.docx]

**
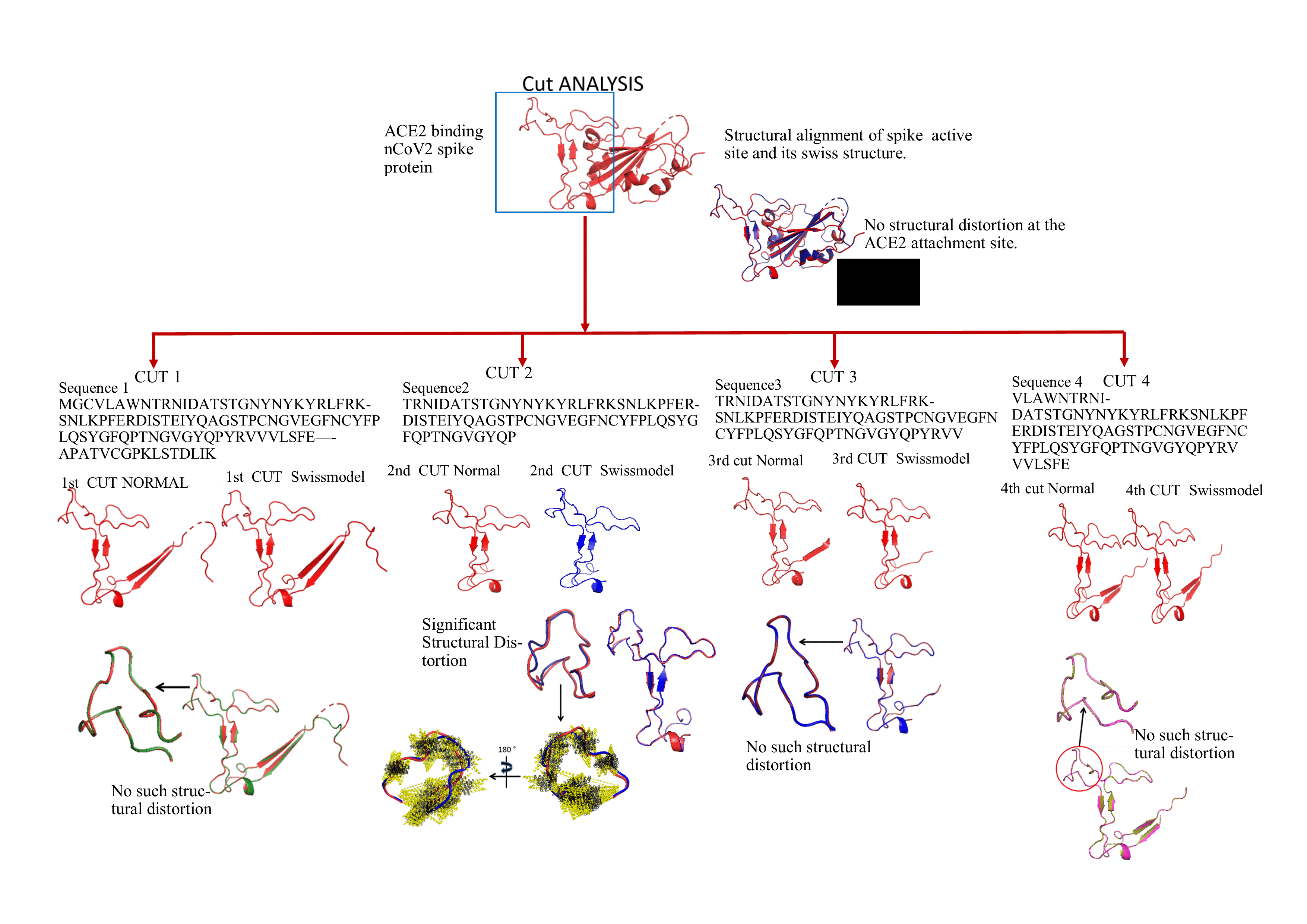
S Figure 1**: Different Cut site analysis of SARS COV-2 spike glycoprotein

**
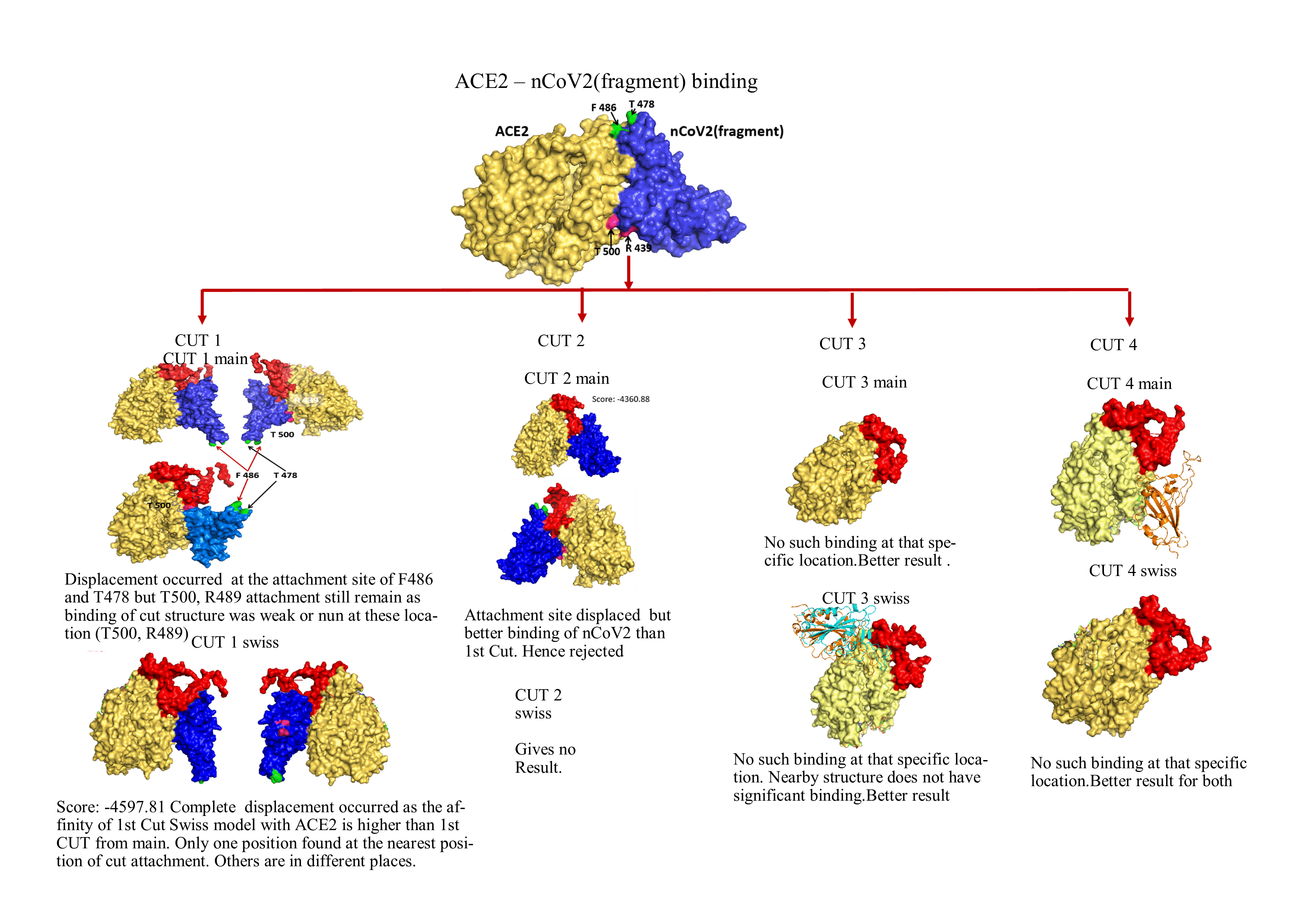
 Figure 2:** Binding interaction site analysis between Cut 1,2,3,4 and ACE Receptor.


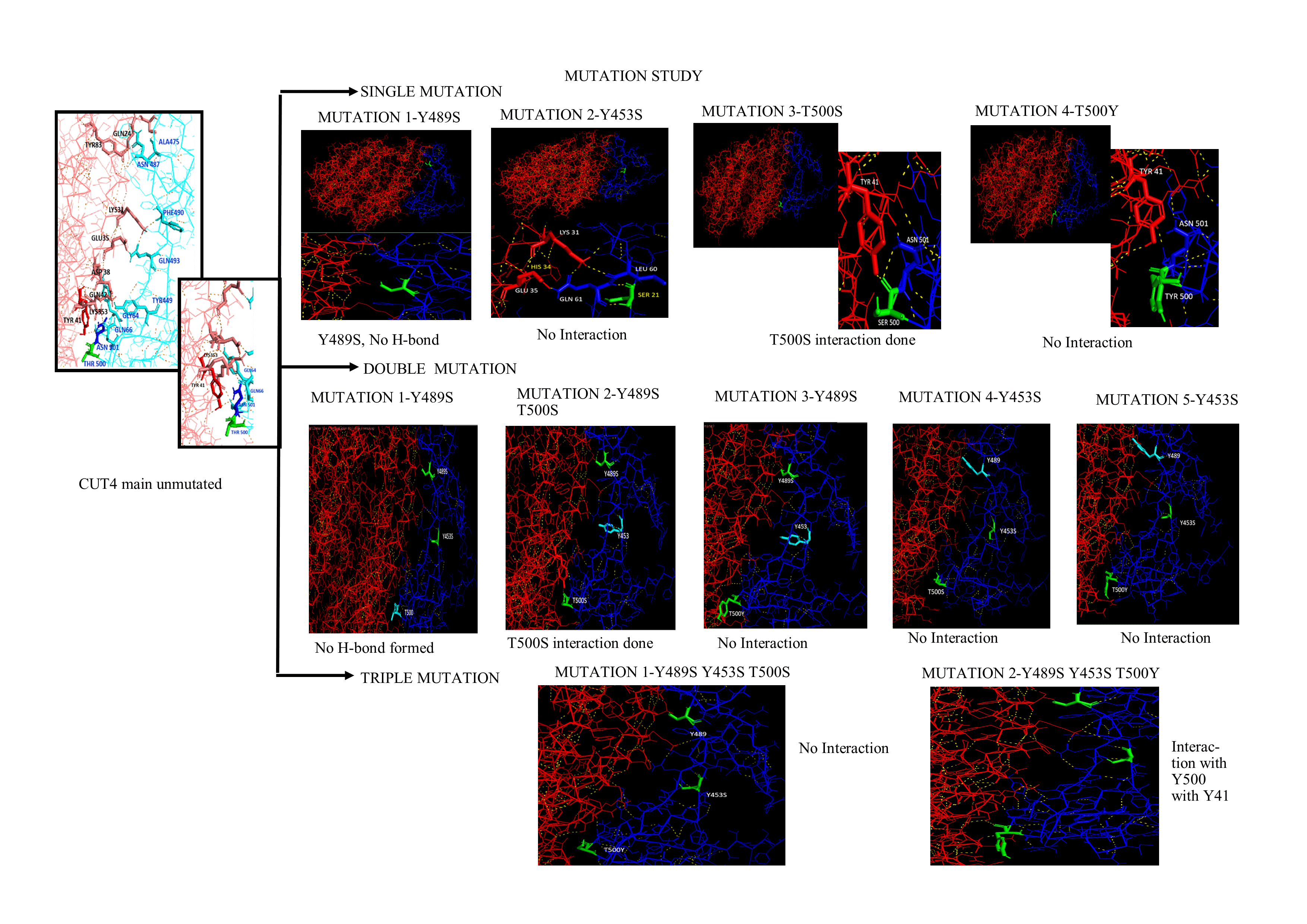
 **S Figure 3**: Depicts the binding sites for Cut 4 Main unmutated with ACE 2 receptor and the resultant interaction in the case of Single Mutation, Double Mutation and Triple mutation induced in Cut 4 with the same.
